# Engineered CD147-Deficient THP-1 Impairs Monocytic Myeloid-Derived Suppressor Cell Differentiation but Maintains Antibody-Dependent Cellular Phagocytosis Function for Jurkat T-ALL Cells with Humanized Anti-CD147 Antibody

**DOI:** 10.3390/ijms25126626

**Published:** 2024-06-16

**Authors:** Thanathat Pamonsupornwichit, Kanokporn Sornsuwan, On-anong Juntit, Umpa Yasamut, Nuchjira Takheaw, Watchara Kasinrerk, Phenphichar Wanachantararak, Kanchanok Kodchakorn, Piyarat Nimmanpipug, Nutjeera Intasai, Chatchai Tayapiwatana

**Affiliations:** 1 Division of Clinical Immunology, Department of Medical Technology, Faculty of Associated Medical Sciences, Chiang Mai University, Chiang Mai 50200, Thailand; thanathat_pamon@cmu.ac.th (T.P.); umpa.yas@cmu.ac.th (U.Y.); nuchjira.t@cmu.ac.th (N.T.); watchara.k@cmu.ac.th (W.K.); 2 Center of Biomolecular Therapy and Diagnostic, Faculty of Associated Medical Sciences, Chiang Mai University, Chiang Mai 50200, Thailand; kanokporn.sornsuwan@cmu.ac.th (K.S.); onanong.j@cmu.ac.th (O.-a.J.); 3 Office of Research Administration, Chiang Mai University, Chiang Mai 50200, Thailand; kanchanok.k@cmu.ac.th; 4 Biomedical Technology Research Center, National Center for Genetic Engineering and Biotechnology, National Science and Technology Development Agency at the Faculty of Associated Medical Sciences, Chiang Mai University, Chiang Mai 50200, Thailand; 5 Dentistry Research Center, Faculty of Dentistry, Chiang Mai University, Chiang Mai 50200, Thailand; phenphichar.w@cmu.ac.th; 6 Department of Chemistry, Faculty of Science, Chiang Mai University, Chiang Mai 50200, Thailand; piyarat.n@cmu.ac.th; 7 Division of Clinical Microscopy, Department of Medical Technology, Faculty of Associated Medical Sciences, Chiang Mai University, Chiang Mai 50200, Thailand

**Keywords:** basigin, leukemia, cancer treatment, immunotherapy

## Abstract

CD147 is upregulated in cancers, including aggressive T-ALL. Traditional treatments for T-ALL often entail severe side effects and the risk of relapse, highlighting the need for more efficacious therapies. ADCP contributes to the antitumor response by enhancing the ability of phagocytic cells to engulf cancer cells upon antibody binding. We aimed to engineer CD147^KO^ THP-1 cells and evaluated their differentiation properties compared to the wild type. A humanized anti-CD147 antibody, HuM6-1B9, was also constructed for investing the phagocytic function of CD147^KO^ THP-1 cells mediated by HuM6-1B9 in the phagocytosis of Jurkat T cells. The CD147^KO^ THP-1 was generated by CRISPR/Cas9 and maintained polarization profiles. HuM6-1B9 was produced in CHO-K1 cells and effectively bound to CD147 with high binding affinity (K_D_: 2.05 ± 0.30 × 10^−9^ M). Additionally, HuM6-1B9 enhanced the phagocytosis of Jurkat T cells by CD147^KO^ THP-1-derived LPS-activated macrophages (M-LPS), without self-ADCP. The formation of THP-1-derived mMDSC was limited in CD147^KO^ THP-1 cells, highlighting the significant impact of CD147 deletion. Maintaining expression markers and phagocytic function in CD147^KO^ THP-1 macrophages supports future engineering and the application of induced pluripotent stem cell-derived macrophages. The combination of HuM6-1B9 and CD147^KO^ monocyte-derived macrophages holds promise as an alternative strategy for T-ALL.

## 1. Introduction

Extracellular matrix metalloproteinase inducer (EMMPRIN), also known as CD147, is expressed in various hematologic and nonhematologic cell types. The significance of CD147 in cancer lies in its pivotal roles in tumor proliferation, invasion, metastasis, and chemoresistance [1]. CD147 is also required for the recruitment and accumulation of myeloid-derived suppressor cells (MDSCs) in tumors [2]. Monocytic MDSCs (mMDSCs) and granulocytic MDSCs are two subgroups of MDSCs that are elevated in late-stage malignancies and decrease T cell immunological activity [3]. In addition to solid tumors, CD147 gene upregulation is demonstrated in hematological malignancies like T-cell acute lymphoblastic leukemia (T-ALL) [4]. Inhibition of CD147 expression using RNA interference reduces cancer aggressiveness [4]. Involvement of matrix metalloproteinase-2 (MMP-2) in acute lymphoblastic leukemia extravasation [5] and elevation of MMP-9 in T cell leukemia patients on T cell infiltration are reported [6]. Additionally, the interaction of CD147 with cyclophilin has contributed to cutaneous T cell lymphoma proliferation and survival [7].

The overexpression of CD147 in cancer cells makes it a promising target for cancer therapy. A number of different approaches are being developed to target CD147, including antibodies and small molecule inhibitors. Anti-CD147 antibodies that hinder cancer cell proliferation and trigger apoptosis have been reported [8,9]. Epitopes within the CD147 domain play distinct biological functions, with the role of the D1 domain being crucial in MMP induction and integrin association [10]. Furthermore, the MMP induction in cancers is associated with CD147 dimerization, resulting in the invasion of cancers [11]. Metuximab targeting the D1 domain epitope significantly overlaps with a region implicated in CD147 dimerization, resulting in the inhibition of HCC invasion and metastasis [12]. Beyond antibody-based interventions, small molecule compounds like SP-8356 [13] and AC-73 [14] have been developed and shown to disrupt CD147 dimerization, offering alternative therapeutic avenues. While antibody-mediated inhibition of CD147 dimerization holds promise in inhibiting cancer metastasis, the internalization of the antibody can further enhance the antitumor effects. HcHAb18-DM1, a novel drug-conjugated antibody, demonstrates this principle by internalizing upon binding to CD147, leading to limiting cancer cell proliferation [15]. Although internalization is an initial process of drug-conjugated antibody therapy, it may limit the efficacy of effector mechanisms like antibody-dependent cellular phagocytosis (ADCP) and antibody-dependent cellular cytotoxicity (ADCC). Recently, the distinctive epitope on CD147, ^31^EDLGS^35^ residing in domain 1 of CD147, was identified as the binding target of mouse monoclonal antibody (mAb), M6-1B9 [16].

Traditional treatments for ALL include chemotherapy, radiation therapy, and stem cell transplantation. In recent years, there has been growing interest in using antibody-based therapies for ALL [17,18] to reduce the side effects of conventional therapy. Recently, self-CAR-T has been applied to target T-ALL [19]. However, the immature T cells in the bone marrow of patients with T-ALL often experience dysfunction regarding their immaturity and mutations [20]. Instead, ADCP relies on macrophages and represents a critical immune effector function that plays a pivotal role in combating cancer through Fc-FcγR interaction on macrophages [21,22]. The enhancement of phagocytic activity through antibody-specific antigens has demonstrated remarkable success in the treatment of cancers, including hematologic malignancies [23] and breast cancer [24].

CD147 is overexpressed in the T-cell acute lymphoblastic cell line (Jurkat); however, the active form with high glycosylation exhibits lower expression compared to other leukemic cell types [25]. This restricted expression of CD147 hampers cancer cell lysis via complement fixation after antibody binding [26]. Accordingly, ADCP should be considered to enhance T-ALL suppression. In addition, macrophages recognize and phagocytose apoptotic cells, but not the viable cells [27]. Moreover, the upregulation of CD47 on Jurkat T cells plays a role in anti-apoptotic function [28]. Hence, ADCP can enhance the clearance of living T-ALL. Recently, a humanized single chain variable fragment (scFv) called HuScFvM6-1B9 specific to CD147 has been generated and characterized [16]. In this study, we further synthesized a fully humanized antibody called HuM6-1B9 or Takatamab for equipping with M-LPS to target Jurkat cells with ADCP function. The M-LPS macrophages were maturated from CD147 knockout THP-1 cells (CD147^KO^ THP-1) to prevent self-ADCP. Apart from phagocytic activity, we also compared certain physiological properties, such as surface molecules and proinflammatory cytokine expressions of induced macrophages from CD147^KO^ and wild type (WT) THP-1 cells. However, as monocytes can develop into mMDSCs, their potential utility for cancer therapy should be considered. In this regard, the polarization of CD147^KO^ THP-1 into mMDSCs was also investigated.

## 2. Results

### 2.1. Production and Purification of HuM6-1B9

**Figure 1:**
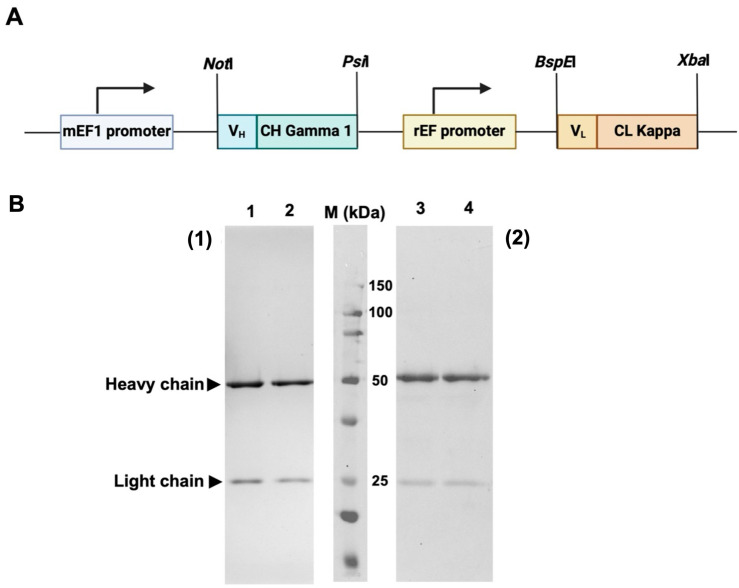
Plasmid construction and determination of purified HuM6-1B9 via SDS–PAGE and Western immunoblotting. (**A**) The diagram shows a pVITRO1-HuM6-1B9-IgG1/κ plasmid composed of an mEF1 promoter, HuM6-1B9 heavy chain, rEF promoter, and HuM6-1B9 light chain. (**B**) Purified HuM6-1B9 was subjected to 12% SDS–PAGE analysis (1) and Western immunoblotting was performed (2). Purified HuM6-1B9 (lanes 1 and 3) and purified human IgG (positive control; lanes 2 and 4) are shown. The protein bands in SDS-PAGE were visualized using PAGE Blue staining. For Western immunoblotting, the nitrocellulose membrane was probed with HRP-conjugated rabbit anti-human IgG (H+L) antibody. The membrane was developed using a chemiluminescent substrate and the signal was quantified by the Biorad ChemiDoc^TM^ MP Imaging System instrument (Bio-rad, Hercules, CA, USA).

The synthetic DNA fragment encoding V_L_C_L_ and V_H_C_H_ of HuM6-1B9 were subcloned into a pVITRO1-Trastuzumab-IgG1/κ plasmid by using *Not*I and *Psi*I for V_H_C_H_, and *BspE*I and *Xba*I for V_L_C_L_ to construct a pVITRO1-HuM6-1B9-IgG1/κ plasmid (Figure 1A). For antibody production, pVITRO1-HuM6-1B9-IgG1/κ was transfected into the Chinese hamster ovarian (CHO-K1) cell line, and the cells carrying the plasmid were selected using hygromycin B. A culture supernatant containing HuM6-1B9 was harvested and subjected to purification using protein L affinity chromatography. The results demonstrated that HuM6-1B9 was successfully generated. The purified recombinant antibody was determined using SDS–PAGE and Western immunoblotting (Figure 1B).

### 2.2. Characterization of HuM6-1B9

**Figure 2:**
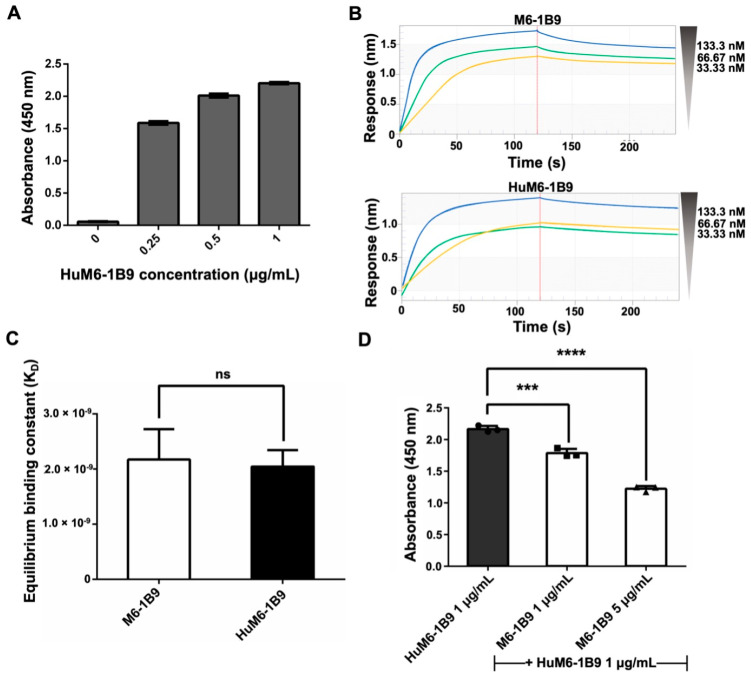
Validation of purified HuM6-1B9 activity, binding affinity, and inhibition binding analysis against CD147-BCCP. (**A**) The ELISA wells were coated with CD147-BCCP, and purified HuM6-1B9 was then added to the well at various concentrations. HRP-conjugated rabbit anti-human IgG antibody was added to detect the binding of HuM6-1B9 to CD147. (**B**) The binding response sensorgrams of M6-1B9 and HuM6-1B9 to the pre-immobilized CD147. The binding curves were fit in a 1:1 fitting mode. (**C**) The K_D_ values of M6-1B9 and HuM6-1B9 were calculated by the ratio of k_d_/k_a_ to determine their binding affinity to CD147 (mean ± SD). Unpaired *t*-test. ns, *p* > 0.05. (**D**) HuM6-1B9 along with M6-1B9 were introduced to the well to compete for binding to CD147-BCCP. The signal was determined by HRP-conjugated rabbit anti-human IgG (H+L) antibody, and the absorbance value at 450 nm was quantified using an ELISA reader. Data are exhibited as mean ± SD. Statistical analysis was determined using a one-way ANOVA. ***, *p* < 0.001 (statistical significance), ****, *p* < 0.0001 (statistical significance).

The indirect ELISA was employed for the validation of the activity of purified HuM6-1B9 against CD147. The absorbance value at 450 nm exhibited a dose-dependent manner (Figure 2A). These data indicated that purified HuM6-1B9 is effectively bound to CD147. Furthermore, biolayer interferometry (BLI) was used to evaluate the binding affinity of HuM6-1B9 compared to its parental antibody (M6-1B9). The sensorgrams of M6-1B9 or HuM6-1B9 towards CD147 are shown (Figure 2B). The K_D_ was equal to 2.17 ± 0.55 × 10^−9^ M and 2.05 ± 0.30 × 10^−9^ M for M6-1B9 and HuM6-1B9, respectively (Figure 2C). These data indicate that HuM6-1B9 retains its activity against CD147, and its binding affinity is comparable to its parental antibody. Furthermore, the competitive ELISA was performed to assess the inhibition binding analysis of HuM6-1B9 and M6-1B9. The results revealed a significant competitive effect of M6-1B9 on the binding of HuM6-1B9 to its target, CD147, in a dose-dependent manner (Figure 2D). These data suggested that HuM6-1B9 interacted with CD147 at the same epitope.

### 2.3. CD147 Expression on WT THP-1, CD147^KO^ THP-1, Jurkat, and CD147^KO^ Jurkat

**Figure 3:**
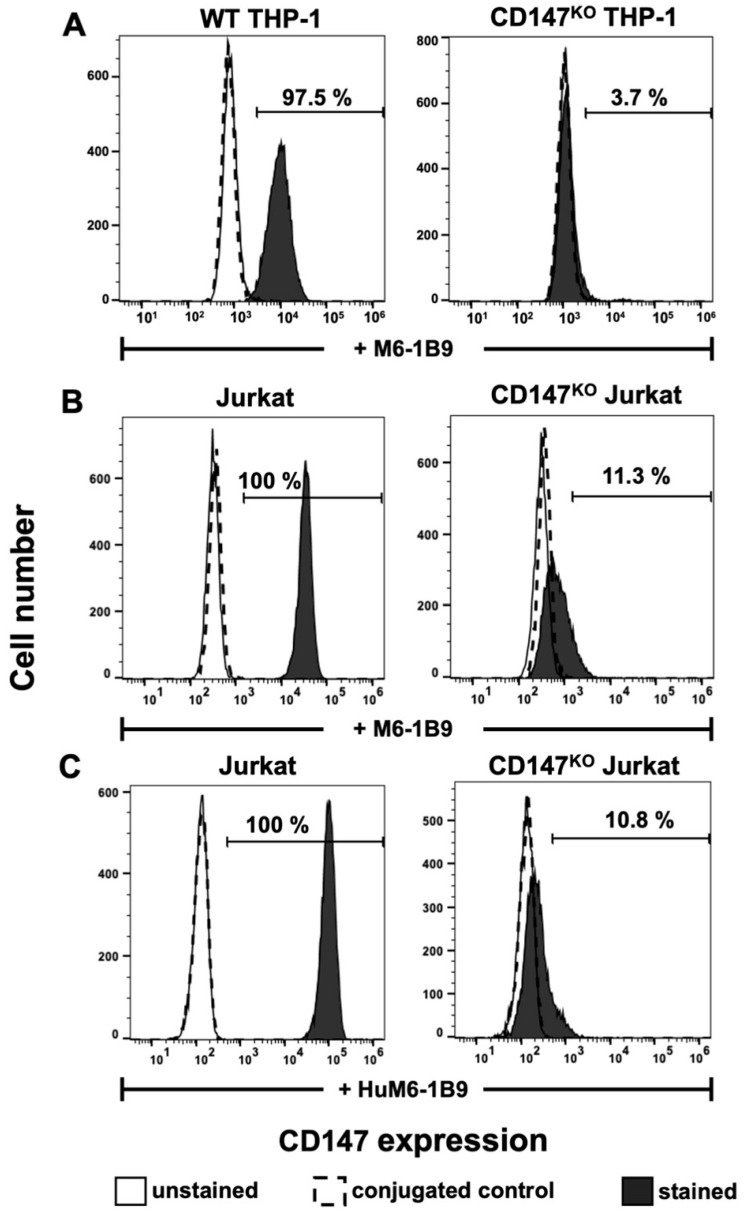
Immunoreactivity of M6-1B9 and HuM6-1B9 toward CD147 on WT THP-1, CD147^KO^ THP-1 cells, Jurkat T cells, and CD147^KO^ Jurkat T cells. (**A**) WT THP-1 or CD147^KO^ THP-1 were incubated with M6-1B9 at 10 μg/mL. (**B**) Jurkat T cells or CD147^KO^ Jurkat T cells were stained with M6-1B9 or (**C**) HuM6-1B9 at 10 μg/mL, and then FITC-conjugated F(ab′)_2_ goat anti-mouse IgG+IgM (H+L) or PE-conjugated goat anti-human IgM/IgG/IgA, F(ab′)_2_ were utilized as secondary antibodies, respectively. The stained cells were analyzed using flow cytometric analysis.

Flow cytometric analysis with M6-1B9 was employed to determine the surface expression of CD147 on WT THP-1, CD147^KO^ THP-1, Jurkat, and CD147^KO^ Jurkat T cells. The results indicated that CD147 was successfully knocked out in CD147^KO^ THP-1 cells (3.7%) in comparison to WT THP-1 (97.5%) (Figure 3A). In addition, the immunoreactivity and binding specificity of HuM6-1B9 against CD147 on Jurkat and CD147^KO^ Jurkat T cells were also performed. The results revealed that both M6-1B9 and HuM6-1B9 recognized CD147 on the cell surface of Jurkat T cells, and insignificantly reacted with CD147^KO^ Jurkat T cells (Figure 3B,C).

### 2.4. Tumor Necrosis Factor-Alpha (TNF-α) and Interleukin-10 (IL-10) Gene Expression, and HLA-DR and CD64 Surface Expression of WT and CD147^KO^ THP-1 Cells

**Figure 4:**
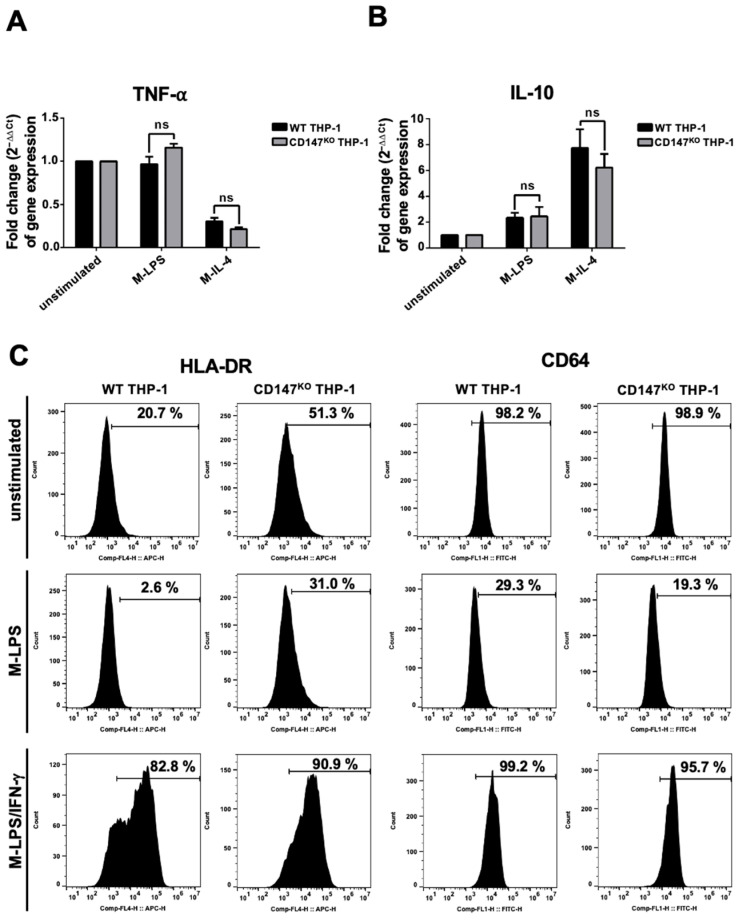
Marker expression of THP-1 and CD147^KO^ THP-1 cells after stimulation. WT and CD147^KO^ THP-1 cells were stimulated with PMA. Following PMA incubation, the cells were incubated with LPS (M-LPS) or IL-4 (M-IL-4). (**A**) The expression levels of *TNF-α*. (**B**) The expression levels of *IL-10*. The fold change of gene expression was calculated using the 2^−ΔΔCt^ method. (**C**) WT and CD147^KO^ THP-1 cells were stimulated to M-LPS or M-LPS/IFN-γ. Cell surface markers, including HLA-DR and CD64, were determined by flow cytometry, and compared to unstimulated cells.

WT or CD147^KO^ THP-1 cells were differentiated into M0-macrophages by phorbol myristate acetate (PMA). After that, the PMA-induced macrophages were polarized to M-LPS or M-IL-4. The induced cells were harvested to determine the gene expression level of *TNF-α* and *IL-10*. There was no significant difference in the expression level of *TNF-α* and *IL-10* between M-LPS and M-IL-4 in both WT and CD147^KO^ THP-1 cells. (Figure 4A,B). These findings suggested that knocking out CD147 from THP-1 did not affect the gene expression of THP-1 cells. Furthermore, the HLA-DR (M1-macrophage phenotype) and FcγRI (CD64) surface expression of WT and CD147^KO^ THP-1-derived M-LPS and M-LPS/IFN-γ were also observed. The results demonstrated that HLA-DR and CD64 expression was downregulated in M-LPS from both WT and CD147^KO^ THP-1 cells. In addition, HLA-DR was upregulated, whereas CD64 was maintained in M-LPS/IFN-γ from both WT and CD147^KO^ THP-1 cells, compared to unstimulated cells (Figure 4C). These data suggested that WT and CD147^KO^ THP-1 cells exhibited similar polarized properties after induction.

### 2.5. Phagocytosis Activity of CD147^KO^ THP-1 Cells

**Figure 5:**
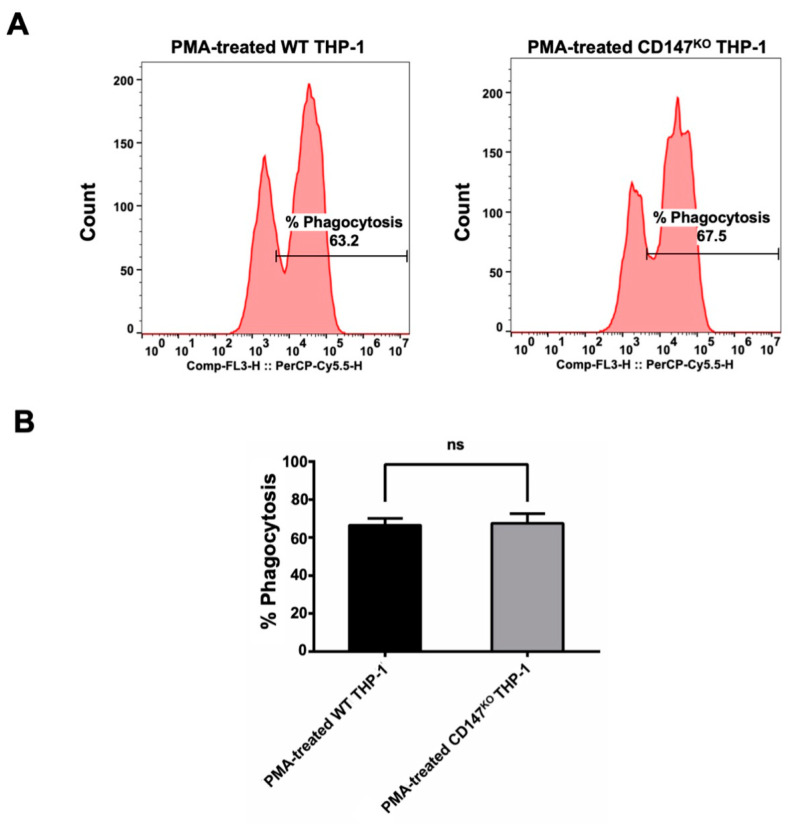
Phagocytosis activity of WT and CD147^KO^ THP-1. (**A**) PMA-treated WT or CD147^KO^ THP-1 cells were harvested after incubation with pHrodo^TM^ Red Zymosan Bioparticles^TM^. The histogram shows the percentage of phagocytosis, which was determined by flow cytometry. (**B**) The bar graph depicts the percentage of phagocytosis observed in WT THP-1 and CD147^KO^ THP-1 cells. The assay was conducted in triplicate and the percentage of phagocytosis was shown as mean ± SD. Statistical analysis was assessed using an unpaired *t*-test. ns, *p* > 0.05 (no statistical significance).

The pHrodo^TM^ Red Zymosan Bioparticles^TM^ (Thermo Fisher Scientific, Waltham, MA, USA) were employed to evaluate the phagocytic function of CD147^KO^ THP-1 cell in comparison to WT THP-1. Both WT and CD147^KO^ THP-1 cells were polarized to M0 macrophages by PMA. Following polarization, the treated cells were incubated with pHrodo^TM^ Red Zymosan Bioparticles^TM^ for 3 h. After incubation, the PMA-treated cells were harvested to determine the percentage of phagocytosis by flow cytometry (Figure 5A). The results indicated that there was no significant difference between the phagocytic function of WT THP-1 and CD147^KO^ THP-1 cells (Figure 5B). This finding suggested that CD147^KO^ THP-1 cells maintain their phagocytic activity.

### 2.6. CD147^KO^ THP-1 Cells Phagocytize CD147-Coated Beads

**Figure 6:**
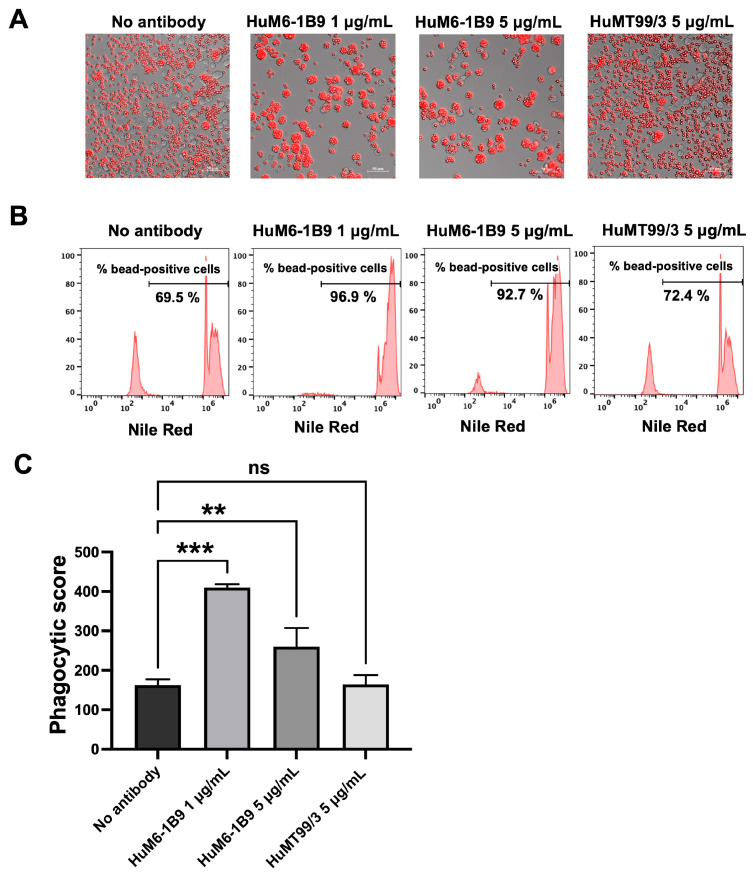
HuM6-1B9 enhances CD147^KO^ THP-1 in phagocytized beads. CD147^KO^ THP-1 cells were co-cultured with CD147-coated streptavidin beads in the absence or presence of HuM6-1B9. (**A**) The phagocytic ability of CD147^KO^ THP-1 cells to uptake CD147-labeled beads was observed under the Zeiss Colibri 7 microscope (200× magnification). (**B**) The histogram indicates the percentage of bead-positive cells, which were analyzed by flow cytometry. (**C**) The graph shows phagocytic scores corresponding to the specific ADCP of HuM6-1B9. The experiment was performed in triplicate and represented as mean ± SD. Statistical analysis was performed using a one-way ANOVA. **, *p* < 0.01 (statistical significance), ***, *p* < 0.001 (statistical significance), ns, *p* > 0.05 (no statistical significance).

The Nile Red streptavidin beads coated with CD147 were incubated at 37 °C for 2 h in the presence or absence of HuM6-1B9. Subsequently, the opsonized beads were co-cultured with CD147^KO^ THP-1 cells for 16 h. The phagocytosed beads within CD147^KO^ THP-1 cells were examined using a fluorescence microscope and the percentage of cells with engulfed beads was quantified using flow cytometry. The results demonstrated that HuM6-1B9 enhanced the phagocytosis of CD147-coated beads by CD147^KO^ THP-1 cells, whereas the absence of HuM6-1B9 or the presence of HuMT99/3 (an irrelevant antibody) resulted in slight bead ingestion by CD147^KO^ THP-1 cells (Figure 6A). Notably, the percentage of bead-positive cells was markedly higher in the presence of HuM6-1B9 compared to the control groups (Figure 6B). In addition, the phagocytic score was calculated and revealed a significant augmentation in phagocytic ability towards CD147-labeled beads when treated with HuM6-1B9, as compared to the control groups (Figure 6C). These findings offer proof that HuM6-1B9 could promote the phagocytic activity of monocytic cells, THP-1, on CD147-labeled beads.

### 2.7. HuM6-1B9 Promotes Phagocytic Activity of CD147^KO^ THP-1 Cells to Engulf Jurkat T Cells

**Figure 7:**
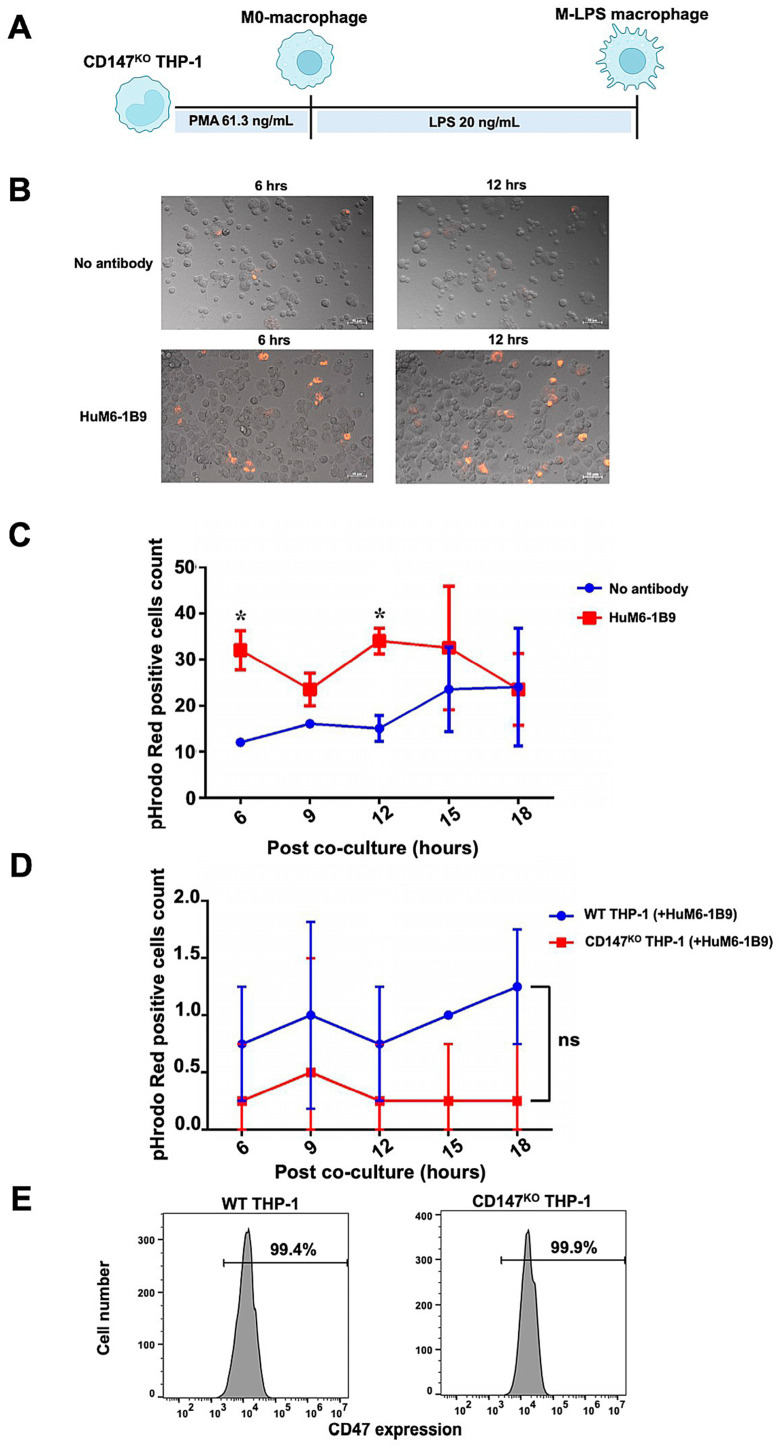
ADCP activity of HuM6-1B9 in Jurkat T cells, and self-ADCP and CD47 surface expression on M-LPS. The pHrodo Red-labeled Jurkat T cells were incubated with M-LPS-like THP-1 cells in the presence or absence of HuM6-1B9. The phagocytosed Jurkat T cells were visualized using a fluorescence microscope. (**A**) The schematic timeline depicted CD147^KO^ THP-1-derivedM-LPS. (**B**) The ability of CD147^KO^ THP-1-derived M-LPS to engulf Jurkat T cells in the absence or presence of HuM6-1B9 at 6 and 12 h. The images were taken under a Zeiss Colibri 7 (200× magnification). (**C**) The number of pHrodo Red-positive cell counts in each time point was shown in the graph, comparing between the absence and presence of HuM6-1B9. (**D**) The graph illustrates the number of pHrodo Red-positive cell counts for determining self-ADCP activity. (**E**) The surface expression of CD47 on WT THP-1 or CD147^KO^ THP-1-derived M-LPS was shown in the histograms. (mean ± SD). Statistical analysis was calculated using a multiple *t*-tests. * *p* < 0.05 (statistical significance). ns, *p* > 0.05 (no statistical significance).

The ADCP function of HuM6-1B9 in Jurkat T cells was assessed. The Jurkat T cells were labeled with pHrodo™ iFL Red STP ester (Thermo Fisher Scientific, Waltham, MA, USA) and subsequently co-incubated with CD147^KO^ M-LPS-like THP-1 macrophages (Figure 7A) at an effector to target (E:T) ratio of 1:2. Phagocytic activity was observed under fluorescence microscope and analyzed with ZEN 2.6 (blue edition) software. After the co-culture of pHrodo Red-labeled Jurkat T cells with CD147^KO^ M-LPS macrophages, minimal phagocytosis of Jurkat T cells by CD147^KO^ M-LPS macrophage cells was observed in the absence of HuM6-1B9. In contrast, the presence of HuM6-1B9 significantly enhances the phagocytic activity of CD147^KO^ M-LPS macrophages in engulfing Jurkat T cells (Figure 7B). The motional movement of the phagocytic CD147^KO^ M-LPS macrophages, those engulfing Jurkat T cells, was demonstrated in the supplemental data (Supplementary Video S1 and S2). The number of pHrodo Red-positive cells revealed that HuM6-1B9 significantly promoted M-LPS cells to phagocytose Jurkat T cells after co-culture for 6 and 12 h (Figure 7C). Moreover, the self-ADCP was not exhibited in the presence of HuM6-1B9 (Figure 7D), indicating that HuM6-1B9 did not promote effector cells eating themselves. The expression of CD47 (a molecule in the “don’t eat me signal”) was also observed in both WT (99.4%) and CD147^KO^ (99.9%) THP-1-derived M-LPS macrophages (Figure 7E). Furthermore, the ADCP function of HuM6-1B9 on peripheral blood mononuclear cells (PBMCs) was also investigated. The results revealed that CD147^KO^ M-LPS macrophage cells did not phagocytose PBMCs in the presence of HuM6-1B9. This observation suggested that HuM6-1B9 did not facilitate the engulfment of PBMCs by CD147^KO^ THP-1-derived M-LPS.

### 2.8. Development of mMDSC-like CD147^KO^ and WT THP-1 Cells

**Figure 8:**
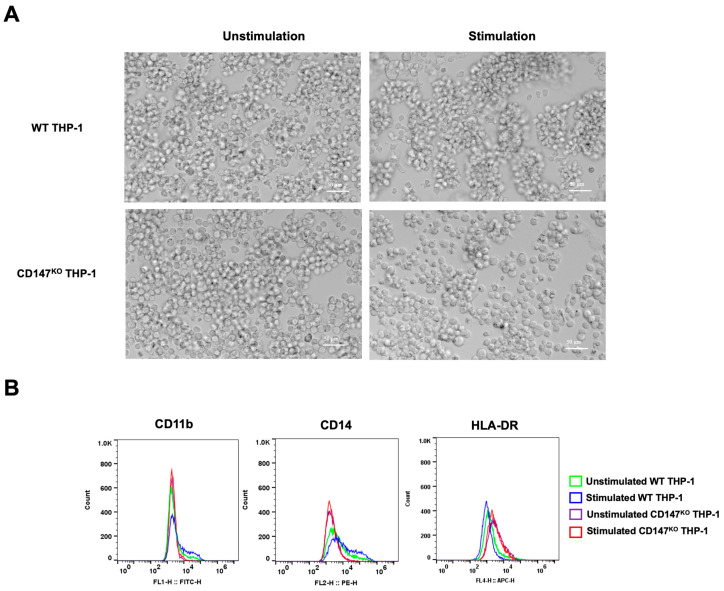
mMDSC differentiation in WT THP-1 and CD147^KO^ THP-1 cells. WT THP-1 and CD147^KO^ THP-1 cells were differentiated into mMDSC-like cells using G-CSF and IL-4. (**A**) At 7 days post-stimulation, cell morphology was observed under inverted microscopy (200× magnification). (**B**) Cells were collected to assess surface expressions of CD11b, CD14, and HLA-DR using flow cytometry. The distributions of cell populations from unstimulated WT THP-1 cells (green line), stimulated WT THP-1 cells (blue line), unstimulated CD147^KO^ THP-1 cells (purple line), and stimulated CD147^KO^ THP-1 cells (red line) are illustrated in the histogram. The data represent two independent experiments.

CD147^KO^ and WT THP-1 were differentiated into mMDSC-like cells using G-CSF and IL-4. After 7 days of stimulation, the morphology of stimulated cells appeared with more clumping than unstimulated cells (Figure 8A). The collected cells underwent analysis for surface marker expression by flow cytometry (Figure 8B). Stimulated WT THP-1 cells exhibited an elevated population of CD11b- and CD14-positive cells in comparison to stimulated CD147^KO^ THP-1 cells. Conversely, the stimulated WT THP-1 cells displayed a lower population of HLA-DR-positive cells. These data imply that the differentiation of CD147^KO^ THP-1 cells into mMDSC-like cells is compromised.

## 3. Discussion

CD147 plays a pivotal role in inducing MMPs, which, in turn, promote angiogenesis in cancer. It also functions as a receptor for cyclophilin A, significantly enhancing cancer cell proliferation [29]. CD147 is not only highly expressed on the cell surface but is also secreted in tumor-derived exosomes [30], promoting the secretion of small extracellular vesicles from cancer stem cells [31], ultimately contributing to tumorigenesis. MAbs targeting CD147 on cancer cells have been developed and shown to possess anti-tumor functions [32]. Recently, the anti-CD147 nanobody-conjugated doxorubicin has been developed for cancer therapies [33]. Furthermore, the anti-CD147 antibody, Metuzumab, has exerted anti-tumor activity by mediating ADCC [34]. However, the ADCP response generated by the anti-CD147 antibody has not yet been reported. Accordingly, the fully humanized anti-CD147 antibody, Takatamab, was synthesized as a partner of engineered CD147^KO^ THP-1 effector cells in ADCP.

The pVITRO1-HuM6-1B9-IgG1/κ plasmid was constructed through subcloning of the V_L_C_L_ and V_H_C_H_ regions of HuScFvM6-1B9 [16] into a pVITRO1-Trastuzumab-IgG1/κ plasmid. This plasmid was designed to produce the Fc portion of the recombinant antibody in the human IgG1 isotype, known for its ability to bind to all subclasses of human FcγR receptors [35] and its potential to stimulate immune-effector functions, including ADCP [35]. Takatamab with suitable codon optimization was produced from the CHO-K1 cell line. The nucleotide sequence coding for variable domains was optimized for expression in CHO-K1 cells [36]. The K_D_ value of Takatamab (2.05 ± 0.30 × 10^−9^ M) demonstrated a comparable degree to its parental antibody, M6-1B9 (2.17 ± 0.55 × 10^−9^ M). Additionally, the K_D_ of Takatamab was higher than that of the HuScFv form, as reported in our previous study [16]. In addition, the epitope recognized by Takatamab was proven to overlap with parental mAb M6-1B9 by competitive ELISA. Whereas ^31^EDGLS^35^ was identified as the epitope for Takatamab, this determinant differs from the region formerly suggested to participate in CD147 dimerization [11]. Consequently, Takatamab is expected to be more accessible for interaction with the epitope, even in the presence of a formed CD147 dimer, thereby providing a therapeutic advantage for ADCP.

Since Takatamab recognizes the reachable epitope on CD147, ADCP was performed to validate the phagocytic function. The CD147^KO^ THP-1 cells were constructed to prevent self-ADCP of M-LPS derived from THP-1. Notably, CD147 has been reported to serve as a gene promoting phagocytosis [37], and its absence may hinder phagocytic activity. The polarization of CD147^KO^ THP-1 cells was investigated and compared with WT THP-1 by examining the expression markers for M1- and M2-like macrophages. Cytokine expression, including TNF-α, and the protein receptor HLA-DR were utilized as markers for M1-like macrophages, while IL-10 served as markers for M2-like macrophages [38]. In our current study, M-LPS exhibited higher *TNF-α* gene expression compared to M-IL-4, while the *IL-10* gene expression level of M-LPS was lower. This suggested that the polarization of M0-macrophages with LPS promoting the M1-like macrophage phenotype, whereas IL-4 supported M0-like THP-1 toward the M2-like phenotype. However, HLA-DR expression, another M1-macrophage marker, was diminished in M-LPS, but expressed higher in M-LPS/IFN-γ. These findings highlight that M-LPS/IFN-γ strongly exhibits a higher M1-like phenotype compared to M-LPS. Although the HLA-DR contributes to antigen presentation, M-LPS was employed in ADCP due to its previously reported ability to induce higher phagocytic activity compared to M-LPS/IFN-γ [39]. The ADCP functional analysis of M-LPS/IFN-γ should be further investigated. Interestingly, CD147^KO^ THP-1 cells retained their ability to polarize into M1- and M2-like macrophages. It is important to note that CD147 expression was reported to synchronize with CD163, leading to an increased infiltration of tumor-associated macrophages [40]. In addition to its primary effect, knocking out CD147 on macrophages may act as a deterrent against TAM infiltrating the tumor microenvironment.

To assess phagocytic function, WT and CD147^KO^ THP-1 cells were differentiated into M0 macrophages through PMA treatment. The internalized activity of PMA-treated WT and CD147^KO^ THP-1 cells did not significantly differ after incubation with pHrodo^TM^ Red Zymosan Bioparticles^TM^. These bioparticles can be used to determine the reactive oxygen species (ROS) secretion in the phagosome of phagocytic cells, based on the emission of pH-sensitive pHrodo Red dye in the acidic phagosome. Our data suggest that knocking out CD147 did not significantly affect the ROS production in PMA-treated THP-1 cells. We further investigated the ADCP function of Takatamab, which demonstrated strongly enhanced phagocytic activity in CD147^KO^ THP-1 cells to phagocytose CD147-labeled polystyrene beads with diameters ranging from 5.0 to 7.9 µm. However, Takatamab did not affect the engulfment of CD147-labeled beads by CD147^KO^ THP-1 cells in a concentration-dependent manner. This observation is consistent with a study conducted by Pacheco et al. in 2013 [41], which depicted that the Fc density on particles has a less significant correlation with internalization activity for particles larger than 2 µm [41].

Monocyte-derived macrophages can be categorized into two distinct types: M1 macrophages (with an antitumor effect) and M2 macrophages (with a protumor effect) [42]. Although both types of macrophages can be found in the tumor microenvironment (TME), macrophages recruited into the TME predominantly exhibit an M2-like phenotype [42]. Interestingly, M1-type macrophages exhibit a higher efficacy in the ADCP mechanism than M2-like phenotype macrophages [43]. Therefore, we aim to polarize CD147^KO^ THP-1 cells into an M1-like macrophage phenotype. This polarization was achieved using LPS or IFN-γ independently or in combination as described previously [39,44]. The phagocytic function of CD147^KO^ THP-1-derived M-LPS was enhanced by Takatamab to promote ADCP responses to Jurkat T cells. Likewise, Deckert et al. (2014) demonstrated that employing a humanized antibody with human IgG1/κ isotype targeting CD38 facilitated the ADCP mechanism in different hematologic malignancies [23]. The data in Figure 7C suggest that maximum phagocytosis in the presence of HuM6-1B9 was achieved after 12 h of co-culture. However, following 15 h of incubation, the levels of phagocytosis between the no antibody and HuM6-1B9 conditions became similar. The reduction in pHrodo Red-positive cells after 12 h of HuM6-1B9 treatment could be attributed to the lysosomal degradation of phagocytosed Jurkat T cells within phagolysosomes. Furthermore, the absence of antibody condition exhibited comparable levels of phagocytosis at 15 h to the HuM6-1B9 treatment, with a slight increase in pHrodo Red-positive cells. This might be due to the apoptotic death of Jurkat T cells during incubation, which can be recognized and cleared by phagocytes through phagocytosis. The self-ADCP was not observed in the polarized M-LPS THP-1 when co-cultured with Takatamab. This phenomenon implied that the expression of CD47, the “don’t eat me” signal, was not affected by disrupting the CD147 expression. Although the expression level of CD64 was reduced in CD147^KO^ THP-1-derived M-LPS, the high affinity of CD64 adequately supports the ADCP response [45]. The absence of ADCP observed with PBMCs despite the effectiveness of Takatamab against CD147-labeled polystyrene beads and Jurkat T cells prompts the consideration for potential applications of Takatamab.

MDSCs exert an immunosuppressive function by inhibiting T cell response, thereby suppressing the host immune response against cancer [46]. Various approaches target MDSCs in cancer, including depleting the MDSC population, blockade of MDSC recruitment, inhibiting the activity of MDSCs, and the differentiation of MDSCs to mature myeloid cells [47]. In this study, we investigated the impact of CD147 on THP-1 in differentiating into mMDSCs by assessing human mMDSC markers (CD11b^+^CD14^+^HLA-DR^low/neg^) [48] after polarization. Interestingly, CD147^KO^ THP-1 cells possess a negative ability to develop into mMDSC-like cells which could attenuate their immunosuppressive properties and suppress their role in tumor progression. Remarkably, CD147 is essential for both attracting and accumulating mMDSCs [2]. Accordingly, the knocking out CD147 from THP-1 could confer a significant advantage, rendering the engineered macrophage as effector cells in treatment strategies for cancer.

## 4. Materials and Methods

### 4.1. Cell Lines

Jurkat T cells, clone E6-1 (ATCC, Manassas, VA, USA), CD147^KO^ Jurkat [16], WT THP-1 (ATCC, Manassas, VA, USA), and CD147^KO^ THP-1 were cultured in the Roswell Park Memorial Institute 1640 (RPMI-1640) medium supplemented with 10% heat-inactivated fetal bovine serum (FBS), 100 U/mL penicillin, 100 μg/mL streptomycin, and 2 mM L-glutamine. CHO-K1 cells were purchased from ATCC (ATCC, Manassas, VA, USA) and maintained in the Iscove’s modified Dulbecco’s medium (IMDM), supplemented with 10% FBS, 100 U/mL penicillin, 100 μg/mL streptomycin, and 2 mM L-glutamine.

### 4.2. Construction of CD147^KO^ THP-1 Cell Line

To establish CD147^KO^ THP-1 cells, the formation of ribonucleoprotein (RNP) complex, including spCas9 (Integrated DNA Technologies, Coralville, IA, USA) and single guide RNA (sgRNA) targeting CD147 was prepared as previously reported [16]. The RNA complex was then transfected into 5 × 10^5^ THP-1 cells using 4D-Nucleofector^TM^ X Kit S (Lonza, Basel, Switzerland) with the FF100 program of the 4D-Nucleofector^TM^ X unit. Following nucleofection, the cells were maintained in a 20% FBS RPMI-1640 medium.

### 4.3. The Construction of a Plasmid Expressing HuM6-1B9 in CHO-K1 Cells

Codon optimization of variable domains from HuScFvM6-1B9 (GenBank accession MW355841.1) for CHO gene expression was performed utilizing the Genscript Codon Optimization Tool. The synthetic DNA fragments encoding *BspE*I-V_L_C_L_-*Xba*I and *Not*I-V_H_C_H_-*Psi*I were digested using their respective appropriate restriction enzymes. Subsequently, the purified fragments were ligated into the pVITRO1-Trastuzumab-IgG1/κ, replacing the resident V_L_C_L_ and V_H_C_H_ regions of trastuzumab [49]. This resulted in the generation of the derived pVITRO1-HuM6-1B9-IgG1/κ. The plasmid was then transformed into *Escherichia coli* (*E. coli*) XL-1 blue stain. Following transformation, three colonies on LB agar supplemented with 100 μg/mL hygromycin B were selected for plasmid miniprep. To confirm the successful insertion, restriction enzyme analysis and DNA sequencing were performed.

### 4.4. Production and Purification of HuM6-1B9

For the large-scale production of HuM6-1B9, CHO-K1 cells carrying pVITRO1-HuM6-1B9-IgG1/κ were propagated in 10% FBS-IMDM containing 500 μg/mL hygromycin B. Subsequently, 1.5 × 10^8^ cells in 15 mL of 10% FBS-IMDM supplemented with 200 μg/mL hygromycin B were inoculated into the cell compartment of a bioreactor. To support cell growth, 1000 mL of 10% FBS-IMDM containing 200 μg/mL hygromycin B was added to the medium compartment. The culture supernatant was collected on day 7 of inoculation. The culture medium was collected, and new medium was added every 7 days. The collected culture medium containing HuM6-1B9 was pooled and purified using HiTrap^TM^ Protein L affinity chromatography (Cytiva, Uppsala, Sweden).

### 4.5. Production of Biotinylated Human CD147

The production of biotinylated human CD147 in vitro was performed as previously described [16]. Briefly, crude lysate proteins consisting of CD147-BCCP were purified using Strep-Tactin^®^XT 4Flow^®^ resin (IBA LifeSciences, Göttingen, Germany). The purified CD147-BCCP was subsequently subjected to biotinylation using an EZ-Link^TM^ Sulfo-NHS-Biotin kit (Thermo Fisher Scientific, Waltham, MA, USA). The CD147-BCCP was labeled with biotin in a 1:5 molar ratio (CD147-BCCP: biotin). After labeling, biotinylated CD147-BCCP was kept at −80 °C for further application.

### 4.6. Binding Assay of HuM6-1B9 by Indirect ELISA

To determine the binding activity of HuM6-1B9 against CD147, the microtiter wells were immobilized with 50 μL of 1 μg/mL human CD147-BCCP in a moist chamber at 4 °C overnight. Subsequent steps were carried out at room temperature. The coated wells were washed three times with washing buffer (0.05% Tween-20 in PBS), followed by a blocking buffer containing 2% bovine serum albumin (BSA) in 0.05% Tween-20 in PBS for 1 h. After washing, purified HuM6-1B9 at various concentrations was added to the wells and incubated for 1 h. Next, the microtiter plate was washed three times and incubated with 50 μL of HRP-conjugated rabbit anti-human IgG antibody (Thermo Fisher Scientific, Waltham, MA, USA) at a 1:3000 dilution for 1 h. The TMB substrate was used to develop the reaction, and the reaction was then halted using 1 N HCl. The absorbance at 450 nm was measured using an ELISA reader.

### 4.7. Binding Affinity of HuM6-1B9 against CD147 Compared to M6-1B9 by BLI

To assess the binding affinity of HuM6-1B9 toward human CD147 after CDR grafting, the BLI technique was employed. The kinetics of HuM6-1B9 and M6-1B9 were evaluated using a ForeteBio Octet K2 instrument (Fremont, CA, USA). The experimental procedures were conducted in 200 μL/well of assay buffer (0.1% BSA in 0.02% Tween-20 in PBS) at 30 °C. Human CD147, tagged with His (Sino Biological, Beijing, Chiana) was immobilized onto the surface of anti-penta HIS biosensors (HIS1K) (Sartorius, Goettingen, Germany) at 50 μg/mL. Subsequently, the biosensors were subjected to a washing step with the assay buffer. The pre-immobilized biosensors were immersed in solutions of HuM6-1B9 or M6-1B9 at various concentrations to initiate the association phase, thereby generating the corresponding association signals. The dissociation of the antibody–antigen interaction was monitored by immersing biosensors back into the assay buffer. The fitting curves were analyzed using a 1:1 fitting mode, as determined by Octet Data analysis 9.0 software. The equilibrium constant (K_D_) was calculated using the ratio k_d_/k_a_.

### 4.8. Inhibition Analysis of HuM6-1B9 with Mouse Anti-CD147 mAb by Competitive ELISA

Inhibition binding analysis of HuM6-1B9 was investigated using competitive ELISA. The microtiter plate was coated with 50 μL of CD147-BCCP at a concentration of 1 μg/mL and incubated overnight at 4 °C in a moist chamber. Subsequently, the coated wells were incubated with a blocking buffer consisting of 2% BSA in 0.05% Tween-20 in PBS to block non-specific binding proteins. After three washes, 1 μg/mL HuM6-1B9 with or without M6-1B9 at 1 and 5 μg/mL were added to the wells and incubated for 1 h. The microtiter wells were then washed three times and incubated with HRP-conjugated rabbit anti-human IgG antibody at a 1:3000 dilution. The reaction was developed using 50 μL of the TMB substrate, and the absorbance at 450 nm was determined using an ELISA reader after adding 1 N HCl.

### 4.9. Flow Cytometric Analysis on WT THP-1, CD147^KO^ THP-1 Cells, Jurkat, and CD147^KO^ Jurkat

To assess the success of the knocking out CD147 on THP-1, the Fc receptors on WT THP-1 and CD147^KO^ THP-1 cells were blocked using 10% human AB serum on ice for 30 min. Subsequently, cells were stained with M6-1B9 at a final concentration of 10 μg/mL, followed by FITC-conjugated F(ab′)_2_ goat anti-mouse IgG+IgM (H+L) (Immunotools, Friesoythe, Germany) at a 1:10 dilution. Furthermore, the immunoreactivity and binding specificity of HuM6-1B9 toward CD147 on Jurkat and CD147^KO^ Jurkat T cells [16], compared to its parental antibody (M6-1B9), were also performed. The Jurkat and CD147^KO^ Jurkat T cells were harvested and washed three times using a FACS buffer. The cells were then incubated with 10% FBS for 30 min on ice to block their Fc receptors. Next, 50 μL of purified HuM6-1B9 or M6-1B9 was added at a final concentration of 10 μg/mL and incubated on ice for 30 min. The cells were then washed twice with a FACS buffer and subsequently incubated on ice for 30 min with PE-conjugated goat anti-human IgM/IgG/IgA, F(ab′)_2_ (Merck Millipore, Darmstadt, Germany) at a 1:250 dilution or FITC-conjugated F(ab′)_2_ goat anti-mouse IgG+IgM (H+L) at a 1:10 dilution, respectively. Finally, the cells were washed three times using a FACS buffer and fixed with 1% paraformaldehyde in PBS. The stained cells were analyzed using a BD Accuri C6 plus instrument (BD Biosciences, Franklin Lakes, NJ, USA) and FlowJo software version 10.8.2.

### 4.10. Analysis of TNF-α and IL-10 mRNA Expression in LPS- or IL-4-Induced WT and CD147^KO^ THP-1 Cells

*TNF-α* and *IL-10* were used as target genes to determine the physiological properties of WT THP-1 and CD147^KO^ THP-1 cells. A real-time reverse transcription polymerase chain reaction (real-time RT-PCR) was utilized to analyze *TNF-α* and *IL-10* gene expression. WT THP-1 or CD147^KO^ THP-1 cells were differentiated into M0-like macrophages *via* incubation with 61.3 ng/mL of PMA for 6 h [44]. After incubation, the PMA-containing medium was removed and replaced by 20 ng/mL of LPS for 48 h [39] to produce M-LPS macrophages or 20 ng/mL IL-4 for 48 h to obtain M-IL-4 macrophages [44]. The total RNA was extracted from the cells using RNeasy Mini Kit, and subsequently converted to cDNA using the SuperScript^TM^ III First-Strand Synthetic System (Thermo Fisher Scientific, Carlsbad, CA, USA). The cDNA was served as a template to determine the mRNA expression of *TNF-α* and *IL-10*, with *GAPDH* used as a housekeeping gene control. The relative fold gene expression of the stimulated cells and unstimulated cells was calculated using the 2^−ΔΔCt^ method.

### 4.11. Comparison of Polarization Property of CD147^KO^ and WT THP-1 Cells

To assess the polarization property, CD147^KO^ or WT THP-1 cells were differentiated to M0-macrophages using 61.3 ng/mL of PMA. Following incubation, the PMA-containing medium was discarded and replaced with a combination of 10 ng/mL LPS and 5 ng/mL IFN-γ for 18 h to derive M-LPS/IFN-γ [44]. The polarized M-LPS was derived according to the protocol described above. To determine the surface expression of HLA-DR and CD64, the induced cells were stained with APC-conjugated anti-HLA-DR antibody (ImmunoTools, Friesoythe, Germany) or mouse anti-human CD64, clone 10.1 (Thermo Fisher Scientific, Waltham, MA, USA) for 30 min on ice after blocking their Fc receptors. FITC-conjugated F(ab’)_2_ goat anti-mouse IgG+IgM (H+L) was used as a secondary antibody. The HLA-DR and CD64 expression of induced cells was determined by flow cytometry and analyzed by FlowJo software version 10.8.2.

### 4.12. Phagocytosis Activity of CD147^KO^ THP-1

CD147^KO^ or WT THP-1 cells were plated at an amount of 5 × 10^5^ cells/well in a 24-well plate and stimulated with 100 ng/mL PMA. The cells were incubated at 37 °C and 5% CO_2_ for 48 h. Following incubation, the PMA-treated cells were washed twice with an RPMI-1640 medium. The treated cells were incubated with a 1:10 dilution of pHrodo^TM^ Red Zymosan Bioparticles^TM^ in Hank’s Balance Salt Solution (HBSS) for 3 h. The cells were subsequently harvested and analyzed by flow cytometry.

### 4.13. Bead Conjugation and ADCP Assay

Streptavidin-coated fluorescent Nile Red polystyrene beads (Spherotech, Lake Forest, IL, USA) were conjugated with biotinylated CD147 in a solution containing 1% BSA in PBS. Excess biotinylated protein was subsequently removed by washing with 1% BSA in PBS. Following this, the biotinylated CD147-coated beads were incubated with various concentrations of HuM6-1B9 at 37 °C for 2 h. The opsonized beads were subjected to wash three-times using 1% BSA in PBS and subsequently resuspended in a complete RPMI-1640 medium. For ADCP assay, 50 μL of 2.5 × 10^4^ CD147^KO^ THP-1 cells in a flat bottom 96-well plate were incubated with 5 × 10^5^ of the opsonized beads at 5% CO_2_, 37 °C for 16 h. Subsequently, the phagocytic ability and the percentage of phagocytosed beads were determined by fluorescence microscope and flow cytometry, respectively. The phagocytic score was calculated by multiplying the geometric mean fluorescent intensity (gMFI) of phagocytosed beads by the percentage of phagocytosed beads. This calculated value was then divided by 10^6^.

### 4.14. ADCP Functional Analysis of HuM6-1B9 on Jurkat T Cell and Self-ADCP Determination

Ten thousand CD147^KO^ THP-1 cells were polarized to M-LPS macrophages as described above. Target cells, consisting of 2 × 10^6^ Jurkat T cells were labeled with 0.5 µM pHrodo™ iFL Red STP ester. These labeled target cells were co-cultured with effector cells at a 1:2 (E:T) ratio in the presence or absence of 20 µg/mL HuM6-1B9. Live cell imaging was performed using Zeiss Colibri 7 for 24 h with 30 min intervals.

For self-ADCP determination, the polarized M-LPS of WT THP-1 or CD147^KO^ THP-1 cells at 1 × 10^4^ cells were labeled with 0.5 µM pHrodo™ iFL Red STP ester in the presence of HuM6-1B9 at 20 µg/mL. Zeiss Colibri 7 was used for live cell imaging with 30 min intervals for 24 h.

### 4.15. Determination of HuM6-1B9 Mediated ADCP on PBMCs

PBMCs from healthy donors, as normal target cells, were isolated using Ficoll–Hypaque density gradient centrifugation. The PBMCs at 2 × 10^6^ cells/mL were labeled with 0.5 μM pHrodo™ iFL Red STP ester. Subsequently, the labeled PBMCs were co-cultured with CD147^KO^ THP-1-derived M-LPS at an E:T ratio of 1:2 in the presence or absence of HuM6-1B9. The ADCP functional analysis was observed through live cell imaging using Zeiss Colibri 7 for 24 h with 30 min intervals.

### 4.16. Evaluation of Expression of CD47 in Induced WT and CD147^KO^ THP-1 Cells

WT THP-1 or CD147^KO^ THP-1 cells were stimulated to M-LPS, as described above. The cells were then collected using Accutase solution and washed three times with a FACS buffer. After blocking Fc receptors with 10% human AB serum on ice for 30 min, the cells were stained with mouse anti-human CD47 antibody, clone B6H12 (Thermo Fisher Scientific, Waltham, MA, USA) for 30 min on ice. FITC-conjugated F(ab’)_2_ goat anti-mouse IgG+IgM (H+L) was used as a secondary antibody. The stained cells were acquired using the BD Accuri C6 plus instrument and analyzed using the FlowJo software version 10.8.2.

### 4.17. CD147^KO^ and WT THP1-Derived mMDSC Differentiation and Immunophenotyping

To generate mMDSC-like cells, CD147^KO^ and WT THP-1 cells were stimulated with G-CSF and IL-4 [50]. CD147^KO^ or WT THP-1 cells were plated at 5 × 10^5^ cells/well in a 12-well cell culture plate. Cells were incubated with 100 ng/mL of G-CSF (PeproTech, New Jersey, USA) and 20 ng/mL of IL-4 (Immunotools, Friesoythe, Germany) in an RPMI-1640 medium supplemented with a 10% heat-inactivated FBS for 7 days. Fresh medium supplemented with G-CSF and IL-4 were replaced on 3 and 5 days of incubation.

After differentiation, cells were harvested and washed twice with PBS. Cells were incubated in a 10% human AB serum on ice for 30 min. After this incubation, cells were labeled with the following antibodies: FITC-conjugated mouse anti-human CD11b (Immunotools, Friesoythe, Germany), PE-conjugated mouse anti-human CD14 (Immunotools, Friesoythe, Germany), and APC-conjugated mouse anti-human HLA-DR (Immunotools, Friesoythe, Germany), and then incubated on ice for 30 min. The cells were washed three times with a FACS diluent. The stained cells were then determined by flow cytometry using BD Accuri^TM^ C6 plus and analyzed by FlowJo software version 10.8.2.

## 5. Conclusions

CD147^KO^ THP-1 cells maintain their physiological properties and demonstrate efficient phagocytosis of Jurkat T cells when co-administered with Takatamab. Notably, the differentiation into mMDSCs is restricted in CD147^KO^ THP-1 cells. The synergistic effect of CD147^KO^ THP-1 cells and Takatamab suggests an alternative treatment for T-ALL patients.

## Data Availability

All data generated or analyzed during the current study are available from the corresponding author upon reasonable request.

## Supplementary Materials

The following supporting information can be downloaded at: https://www.mdpi.com/article/10.3390/ijms25126626/s1.
